# Cell Wall Modifications during Conidial Maturation of the Human Pathogenic Fungus *Pseudallescheria boydii*

**DOI:** 10.1371/journal.pone.0100290

**Published:** 2014-06-20

**Authors:** Sarah Ghamrawi, Gilles Rénier, Patrick Saulnier, Stéphane Cuenot, Agata Zykwinska, Bas E. Dutilh, Christopher Thornton, Sébastien Faure, Jean-Philippe Bouchara

**Affiliations:** 1 L’UNAM Université, Université d’Angers, Groupe d’Etude des Interactions Hôte-Pathogène EA 3142, Angers, France; 2 Laboratoire de Parasitologie-Mycologie, Centre Hospitalier Universitaire, Angers, France; 3 L’UNAM Université, University d’Angers, INSERM U646, Angers, France; 4 L’UNAM Université, Université de Nantes, Institut des Matériaux Jean Rouxel, Nantes, France; 5 Centre for Molecular and Biomolecular Informatics, Radboud University Medical Centre, Nijmegen, The Netherlands; 6 Department of Marine Biology, Institute of Biology, Federal University of Rio de Janeiro, Rio de Janeiro, Brazil; 7 Department of Biosciences, University of Exeter, Biosciences, Exeter, United Kingdom; 8 L’UNAM Université, INSERM U1063, Angers, France; Louisiana State University, United States of America

## Abstract

Progress in extending the life expectancy of cystic fibrosis (CF) patients remains jeopardized by the increasing incidence of fungal respiratory infections. *Pseudallescheria boydii* (*P. boydii*), an emerging pathogen of humans, is a filamentous fungus frequently isolated from the respiratory secretions of CF patients. It is commonly believed that infection by this fungus occurs through inhalation of airborne conidia, but the mechanisms allowing the adherence of *Pseudallescheria* to the host epithelial cells and its escape from the host immune defenses remain largely unknown. Given that the cell wall orchestrates all these processes, we were interested in studying its dynamic changes in conidia as function of the age of cultures. We found that the surface hydrophobicity and electronegative charge of conidia increased with the age of culture. Melanin that can influence the cell surface properties, was extracted from conidia and estimated using UV-visible spectrophotometry. Cells were also directly examined and compared using electron paramagnetic resonance (EPR) that determines the production of free radicals. Consistent with the increased amount of melanin, the EPR signal intensity decreased suggesting polymerization of melanin. These results were confirmed by flow cytometry after studying the effect of melanin polymerization on the surface accessibility of mannose-containing glycoconjugates to fluorescent concanavalin A. In the absence of melanin, conidia showed a marked increase in fluorescence intensity as the age of culture increased. Using atomic force microscopy, we were unable to find rodlet-forming hydrophobins, molecules that can also affect conidial surface properties. In conclusion, the changes in surface properties and biochemical composition of the conidial wall with the age of culture highlight the process of conidial maturation. Mannose-containing glycoconjugates that are involved in immune recognition, are progressively masked by polymerization of melanin, an antioxidant that is commonly thought to allow fungal escape from the host immune defenses.

## Introduction

There has been an increase in the incidence of human infections due to fungi in the *Pseudallescheria/Scedosporium* complex (*P. boydii*, *Pseudallescheria apiosperma* (anamorph: *S. apiospermum*), *Scedosporium aurantiacum*, *Scedosporium dehoogii* and *Pseudallescheria minutispora*) over recent years [1], [2], [3], [4]. Infections range from localized, such as subcutaneous mycetoma in immunocompetent individuals, to disseminated infections in immunocompromised patients.

In addition, *P. boydii* and the closely related species *S. apiospermum* are the most common species recovered from the respiratory tract of patients with cystic fibrosis [5]. The mechanisms of adherence and establishment of an infection by these fungi in the lung are still largely unknown. It is thought that the infection process in the respiratory tract starts by inhalation and adhesion of airborne conidia that differentiate into hyphae, with both processes mediated by the spore cell wall since that acts as the interface between the fungus and lung tissues.

Adherence is governed by two types of mechanisms, specific receptor-ligand and/or non-specific cellular interactions [6]. Depending on the fungus, specific interactions can involve polysaccharides (mannose polymers [7], glucans or galactosaminogalactan [8]), proteins or glycoproteins bound to the cell wall through covalent or non-covalent bonds (ex. hydrophobins [9], [10] or glycosylphosphatidylinositol-anchored proteins like Pwp7p and Aed1p adhesins of *Candida glabrata* [11] and *CspA* of *A. fumigatus*). Non-specific interactions involve cell surface properties such as electrostatic charge and surface hydrophobicity which reflect the biochemical composition of the cell wall [12]. The importance of such properties has been demonstrated in several fungal models including yeasts [13], [14] and filamentous fungi. Deletion of the *medA* gene in *A. fumigatus, for example,* leads to a modification in the surface physical properties along with impaired adherence to epithelial cells and reduced virulence [15].

Escaping recognition and destruction by the immune system is another challenge for fungal pathogens. In *Aspergillus fumigatus,* rodA hydrophobin contributes to fungal viability *in vivo* by masking fungal pathogen-associated molecular patterns (PAMPs), thus preventing recognition by Dectin-1 and Dectin-2 [16]. Other fungal pathogens, like *Pneumocystis jirovecii* or *Cryptococcus neoformans* have been shown to evade immunosurveillance either by changing the expression of major surface glycoproteins [17] or by means of a capsule that cover the antigenic components of infective propagules and modulate the immune response respectively [18]. Melanin is an additional virulence factor employed by many fungi in order to resist phagocytosis and cellular damage secondary to nitrogen- or oxygen-derived radical attack. Fungal melanin has been reported to limit complement activation, and confer resistance to antimicrobial agents [19]. Modification or inhibition of the expression of melanin or rodA hydrophobins has repercussions on the cell surface physical properties in fungi.

In *P. boydii*, only a limited number of cell wall constituents have been characterized so far including peptidorhamnomannans (PRM) and alpha-glucans [20]. PRMs were demonstrated to play a role in the adhesion of conidia to HEp2 epithelial cells *in vitro* [21] as well as activation of Toll-like receptor (TLR) 4, responsible for cytokine induction in macrophages [22]. Alpha-glucans were shown to partially elicit phagocytosis and to induce secretion of inflammatory cytokines in a mechanism involving CD14, TLR 2 and MyD88 [23]. On the hyphal wall, an acid and alkaline phosphatase activity was also detected [24], as well as ceramide monohexosides suggested to be critical for fungal differentiation [25].

In this study, we investigate temporal changes in the physical properties of the conidial wall of *P. boydii*, and we study the presence of rodlet structures and melanin as putative virulence factors in the pathogen.

## Materials and Methods

### Strain and Culture Conditions

The filamentous fungus *Pseudallescheria boydii* IHEM 15155 (subgroup *P. ellipsoidea*), isolated in 1990 from a bronchial aspirate in Angers University Hospital, was used throughout this study. According to the classification of Gilgado *et al.* [18], [3], [4], this strain was identified after sequencing the internal transcribed spacer (ITS) regions 1 and 2 of ribosomal RNA genes (NCBI accession number EF441725) and the TUB region of the beta tubulin gene (KJ566742).

The strain was routinely maintained on YPD (0.5% w/v yeast extract, 2% w/v glucose, 1% w/v peptone, 0.05% w/v chloramphenicol) agar plates. Conidia were harvested from cultures grown for 5, 9 or 14 days at 37°C by flooding the agar surface with sterile water followed by filtration on a 20-µm pore size nylon filter. Conidia were washed twice with Milli-Q™ water, centrifuged at 5000 g for 5 min at 4°C, and finally counted with a hemocytometer.

For some experiments, the fungus was grown in the presence of 1,8-dihydroxynaphthalene (DHN) melanin inhibitors (tricyclazol, pyroquilon or carpropamid) or 3,4-dihydroxyphenylalanin (DOPA) melanin inhibitors (tropolone, kojic acid or glyphosate). Inhibitors were diluted in ethanol and incorporated into YPD agar to a final concentration of 20 µg/ml, according to Cunha *et al.* [26]. All inhibitors were purchased from Sigma-Aldrich (St. Louis, MI, USA).

### Fluorescence Studies

Freshly harvested conidia (10^8^) were washed once with Tris buffer (0.5 mM Tris, 100 mM NaCl, 1 mM CaCl_2_, 1 mM MgCl_2_, pH 7.0), pelleted by centrifugation, and then resuspended in fluorescein isothiocyanate (FITC)-labeled lectins (Sigma): concanavalin A (Con A), Wheat Germ Agglutinin (WGA), or Peanut Agglutinin (PNA) at a final concentration of 100 µg/ml in Tris buffer. After 30 min of incubation at 37°C with continuous agitation, conidia were washed three times in Tris buffer and then fixed in 1% formaldehyde (v/v) in phosphate buffered saline 0.15 M pH 7.2 (PBS). For controls the fluorescent lectin was omitted or the condia were incubated in the presence of an excess (0.2 M) of methyl α-D-mannopyranoside, N-acetyl glucosamine (GlcNac) or galactose for Con A, WGA and PNA respectively, prior to the addition of the FITC-conjugated lectin. Samples were observed with a fluorescence microscope (Leica DMR) using immersion oil at 1000x.

After labeling with FITC-Con A, fluorescence intensity was also quantified by flow cytometry on a FACSCanto™ II cytofluorometer driven by the FACSDiva™ software (BD Biosciences, Pont de Clay, France). The conidia were gated on the forward scatter/side scatter (FSC/SSC) plots. Fifty thousand cells per sample were analyzed and the mean fluorescence intensity was recorded. Flow-Jo software was used for histograms overlays.

### Transmission Electron Microscopy

Conidia were washed once in cacodylate buffer (0.1 M) and pelleted at 5000 g for 5 min at 4°C. After incubation in fixative solution (2.5% (w/v) glutaraldehyde, 2% (w/v) paraformaldehyde, 0.1 M cacodylate buffer) for 24 hours at room temperature under vacuum, they were washed with cacodylate buffer, and incubated for 24 hours in 2% KMnO_4_ in cacodylate buffer at 4°C. After washing, fixed conidia were incubated for 2 hours at room temperature in 2% osmium tetroxide, washed in Milli-Q™ water, and finally dehydrated through a series of ethanol-water solutions (50, 70, 95% ethanol, 2×30 min each) followed by 100% ethanol (3×20 min). The ethanol was finally substituted with propylene oxide (3×20 min). Conidia were impregnated overnight in a propylene oxide-Epon mixture (1∶1 v/v), then embedded in pure Epon for 16 hours and 8 hours. Polymerization was performed at 37°C for 24 hours, then at 45°C for 24 hours and finally at 60°C for 48 hours. Thin sections were counter stained with uranyl acetate and lead citrate in the case of melanin inhibition experiments, otherwise they were directly examined on a JEM-1400 transmission electron microscope (Jeol, Paris, France) operating at 120 kV.

For gold-conjugated Con A labeling, conidia (10^8^) were incubated for 30 min at 37°C with continuous shaking in the presence of 5 nm gold-conjugated Con A (Biovalley, Marne la Vallée, France) diluted 1∶50 in Tris buffer. Then conidia were washed three times in Tris buffer. Two controls were also prepared, by omission of the lectin, or the inclusion of 0.2 M methyl α-D-mannopyranoside in Tris buffer added just prior to the addition of the gold-conjugated lectin. Samples were prepared for TEM as previously indicated without contrasting.

### Surface Physical Properties

#### Zeta-potential

Conidia were washed once in Milli-Q™ water containing 1 mM NaCl, then resuspended in the same solution to a density of 10^6^ conidia/ml. The electrophoretic mobility of cells was measured with a zeta potential analyzer (Zetasizer Nano ZS; Malvern Instruments Ltd, Malvern, UK) before being converted to zeta potentials using the Smoluchowski equation. The measurements, performed at 25°C, were repeated three times with ten to thirty cycles for each measurement. Experiments were repeated three times.

#### Two-phase partitioning

Cell surface hydrophobicity (CSH) was determined essentially as described by Pihet *et al.* [27] with some modifications. Hexadecane (0.5 ml) was dispensed onto a spore suspension (2.5 ml) prepared in PBS (5×10^7^ conidia/ml). A control of 1 ml conidia suspension without hexadecane was prepared and the test samples were prepared in duplicates. Tubes were vortexed for 2 min, allowed to stand at room temperature for 3 min before carefully transferring 1 ml of the aqueous phase (bottom) into a new tube using a Pasteur pipette. The transferred material was vortexed again for 2 min, and the optical density (OD) was read at 405 nm in triplicate on 200-µl aliquots. The hydrophobic index (percentage of conidia that were excluded from the aqueous phase) was calculated using the following equation:
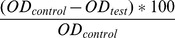



### Atomic Force Microscopy (AFM) Measurements

Conidial suspensions of *P. boydii* with different cell densities were prepared in PBS and 500 µl were added per well in a 24-well plate containing poly-L-lysine (0.1% (w/v) in distilled water, Sigma-Aldrich)-coated 12 mm-diameter glass cover slips prepared according to the manufacturer’s recommendations. Cells were incubated with the coated cover slips for 30 min at 37°C with gentle agitation. Afterwards, the cover slips were washed twice with PBS (5 min each with agitation), then left to dry at ambient temperature and conserved at 4°C before analysis.

The surface of *P. boydii* conidia was imaged using a NanoWizard atomic force microscope (JPK, Berlin, Germany) operating in intermittent contact mode under ambient conditions. A standard rectangular cantilever (Nanosensors NCL-W) was used for imaging, with a free resonance frequency of 165 kHz and a typical spring constant of about 40 N/m. The radius curvature of the tip was ∼10 nm.

### PCR Conditions and Gene Sequencing

#### Genomic DNA extraction

Mycelium from 10 day-old culture in YPD broth was harvested and ground in liquid nitrogen with a mortar and pestle. Intact genomic DNA was obtained by the method of Moller *et al.* [28]. Briefly, the ground mycelium (150–300 mg) was washed three times with methanol containing 0.1% β-mercaptoethanol. Dried pellet was resuspended in 500 µl TES (100 mM Tris pH 8.0, 10 mM EDTA, 2% SDS) containing 5 µl proteinase K (100 µg). After incubation for 1 hour at 60°C, 140 µl NaCl (5 M) and 65 µl of 10% CTAB (cetyltrimethylammoniumbromide) were added to the mixture which was then incubated for 10 min at 65°C. An equal volume of chloroform:isoamylic alcool (24∶1, v/v) was then added. After incubation for 30 min on ice and centrifugation at 20,000 g for 10 min at 4°C, the upper phase was transferred and gently mixed with 0.215 vol (approximately 130 µl) ammonium acetate. After a further 30-min incubation on ice and centrifugation, DNA was precipitated by mixing the supernatant with 0.55 vol isopropanol and immediate cooling on ice before centrifugation for 10 min at 20000 g. The pellet was washed twice with cold 70% ethanol, dried, and resuspended in 50 µl Tris-EDTA (10 mM Tris pH 8.0, 0.1 mM EDTA). The DNA extract was finally treated with RNase.

#### Gene sequencing

The polyketide synthase type I (*PKSI*) and tetrahydroxynaphthalene reductase (*4HNR*) genes were sequenced using degenerate primers listed in Table 1. Primers for *PKSI* were designed from the alignment of *Colletotrichum lagenarium* polyketide synthase (Accession no. D83643) and a homologue of *PKSI* in the genomic sequence database of *P. boydii* (C.R. Thornton, unpublished) using the Multalin program [4] (http://multalin.toulouse.inra.fr/multalin/). Similarly, primers for PCR amplification of an internal fragment of *4HNR* gene were designed from the multi-alignment of four fungal ortholog sequences or their corresponding cDNA: *T4HR1* gene of *Colletotrichum orbiculare* (Accession no. AB661336), *4HNR* gene of *Scedosporium prolificans* strain 3.1 (Accession no. JX861395), 1,3,6,8-tetrahydroxynaphthalene reductase gene of *Magnaporthe grisea* (Accession no. AY846877) and hydroxynaphthalene reductase gene of *Ophiostoma floccosum* (Accession no. AF285781).

**Table 1:** Primers for sequencing genes involved in the melanin synthesis pathway.

Primers*	Primer sequence 5′→3′(**)	Tm
***PKSI*** ** gene sequencing**		
F PKSI.29	ACMAACCAYTCTGCYGA	49°C
R PKSI.29	TAGATWGTATCGCTKGC	
F PKSI.31	GTCGTTTGGGAATGCCTCA	55°C
R PKSI.31	GAGGATCAACGCCAGCCT	
F PKSI.32	GTTATTGTTCAGCTCGGTCTTTG	55°C
R PKSI.32	GTGCTCCGTTGACAACATG	
F PKSI.39	GAACTGATGAAGGCTTGCGGATGTA	60°C
R PKSI.39	GTAGAAGHAATGGCGGAGGCGGC	
F PKSI.40	GCTTAACGAGAAATATCACGCCCAAG	60°C
R PKSI.40	GCTCTCCTTGTGGATTCTGAGGCTC	
F PKSI.41	CTCAGTACCTGCGGCAATCATG	60°C
R PKSI.41	CAGTAGCAGTGGTCGGTCTTCT	
F PKSI.42	CATGTTGTCAACGGAGCACCAC	60°C
R PKSI.42	CTCCTCCCTCAGTCGCCCA	
F PKSI.43	GGCGTTGTGGGTCAAATTAAGTTCC	60°C
R PKSI.43	GTAAGGTCCAGTTGGCTGTCGGCGT	
F PKSI.44	CGCCACCAGCTATACCGAAATCG	60°C
R PKSI.44	CCAAGCGAGCCAAAAGACTAAGAC	
F PKSI.50	CCTCACATACTTCTGTCGGGAG	55°C
R PKSI.50	GGAGCTAAAGTTGGAGAATGCTC	
F PKSI.51	CTCATATTTCAGGTCTGCGGAGAG	55°C
R PKSI.51	GGAATCCATGTGTGTCCAACGA	
***4HNR*** ** gene sequencing**		
F 4HNR.70	GCCGAYATCAGCAAGCC	57°C
R 4HNR.70	CGTAGTGCCASGAGTTCTCG	
***4HNR*** ** primers and adaptor primers for Walking-PCR**		
F1.4HNR.74	CCGCATCATCCTCACCTCTTCC	60°C
F2.4HNR.74	CCACAATCATGCTCTCTACGCCG	60°C
F1.4HNR.83	AGCAGGTTACCGTCAACGCCATC	60°C
F2.4HNR.83	GATCATTGACCAGGGTCTTGCCAAC	60°C
R1.4HNR.75	GGAAGAGGTGAGGATGATGCGG	60°C
R2.4HNR.75	GGACCACACCTCAGTACCCGA	60°C
AP1	GGATCCTAATACGACTCACTATAGGGC	60°C
AP2	AATAGGGCTCGAGCGG	60°C

*Primers carrying the same number represent forward and reverse primers included in the same amplification reaction. Tm of each amplification reaction is indicated in the same row next to the forward primers.

**IUPAC-IUB symbols for nucleotide nomenclature: M = A or C; Y = C or T; W = A or T; K = G or T; and H = A, C or T.

The upstream and downstream regions of the amplified *4HNR* gene fragment were obtained by walking-PCR as described by Siebert *et al.* [29]. Briefly, genomic DNA (2.5 µg) of *P. boydii* IHEM 15155 was digested overnight at 37°C with restriction enzymes *EcoR*V, *Nru*I or *PvU*II according to the supplier’s recommendations (New England Biolabs, Evry, France). Then 10 µl of the obtained fragments (250 ng) were ligated to adaptors (1 pmol) overnight at 14°C; afterwards the ligated products were used as template to amplify by nested PCR the flanking regions of the known gene fragment using outer primers directed towards the adaptor AP1 and the end of the known fragment (indicated by F1 for downstream sequence or R1 for upstream sequence in Table 1). The second step of the nested PCR was then performed using adaptor primer AP2 and inner primers also targeting the known gene fragment (indicated by F2 or R2 in Table 1).

PCR conditions were as follows: 5 min of denaturation at 95°C, followed by 30 cycles of 30 s at 95°C for denaturation, 30 s at 55–60°C for annealing and 1 min at 72°C for elongation, with a final elongation step of 10 min at 72°C. For walking-PCR, the first round was performed as follows: 94°C for 3 min, then 35 cycles (94°C for 30 s, 55°C for 1 min, 72°C for 4 min) and finally 72°C for 15 min. For the second round, PCR conditions were the same except for the number of cycles (25 instead of 35) and duration of final denaturation (30 min instead of 15 min). Amplicons were excised from the gels and purified using NucleoSpin Gel and PCR Clean-up kit (Macherey-Nagel, Hoerdt, France), and then sent for bidirectional Sanger sequencing at GATC Biotech Platform (GATC Biotech AG, Köln, Germany). Sequence analysis was performed using the Translation tool in ExPASy SIB Bioinformatics Resource Portal (http://web.expasy.org/translate/) and The Basic Local Alignment Search Tool (BLAST) in NCBI.

#### Nucleotide sequences accession number

The sequences of the *PKSI* and *4HNR* genes of *P. boydii* IHEM 15155 were deposited in the GenBank database (Accession no. KC440182 and KJ549637 respectively).

### Melanin Extraction and Quantification

#### Electron Paramagnetic Resonance (EPR)

Freshly harvested conidia were washed and resuspended in PBS (150 mM) to a final density of 1.0×10^9^ conidia/ml. Fungal suspensions were aspired in 1 ml syringes and frozen by immersion in liquid nitrogen. EPR spectra were recorded using a MiniScope MS200 EPR spectrometer (Magnettech, Berlin, Germany) operating at X-band. Samples were held in a quartz immersion finger Dewar filled with liquid nitrogen. Experimental parameters were as follows: modulation amplitude 1 G, microwave power 0.1 mW, modulation frequency 100 kHz, microwave frequency 9.5 GHz, number of scans averaged 3. Images were treated using Multiplot 2.0 [30].

#### Melanin extraction

Melanin was extracted according to the method of Gadd [31]. *Pseudallescheria boydii* conidia (5×10^9^) were washed once with MilliQ™ water, then with NaOH (1 M) and centrifuged at 4500 g for 5 min at 4°C. The pellet was mixed with 5 ml NaOH (1 M) and the pigments were extracted by boiling for 20 min followed by autoclaving for 20 min at 115°C. Cell debris were removed by centrifugation at 12000 g for 5 min and the supernatant was acidified to pH 2 with concentrated HCl. Melanin was precipitated after centrifugation at 12000 g for 5 min, then the pellet was washed with MilliQ™ water and finally lyophilized.

#### UV-visible spectrum

Melanin pellets were reconstituted in 1 ml of NaOH 1 M, then diluted to avoid saturation. A standard curve was designed based on the absorption spectra of synthetic DOPA melanin (Sigma) that was solubilized in NaOH 1 M at different concentrations ranging from 0.01 mg/ml to 1 mg/ml. Spectra for extracted and synthetic melanin were recorded in the wavelength range 190–900 nm using a UV-visible spectrophotometer (UV-2600; Shimadzu Scientific Instruments, Columbia, USA).

### Statistical Analysis

Statistical analysis was conducted using a non-parametric test Kruskal–Wallis one-way analysis of variance (ANOVA) or two-way ANOVA with Bonferroni post-hoc test using GraphPad Prism V5.0 software (GraphPad Software, San Diego, CA, USA). *P*-values were considered significant if lower than 0.05.

## Results

### Surface Accessibility of Cell Wall Polysaccharides to Fluorescent Lectins

**Figure 1:**
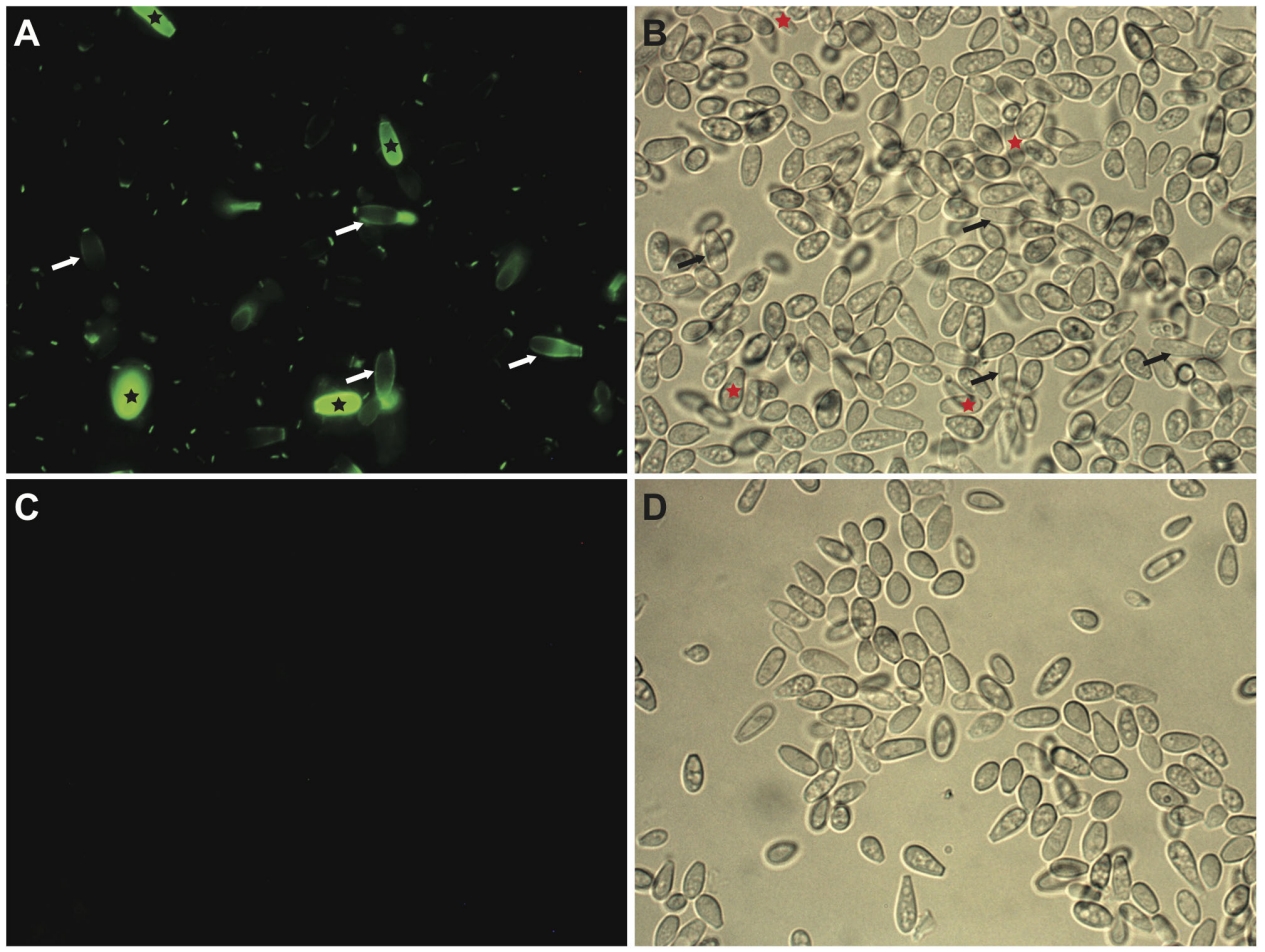
Fluorescence labeling of cell wall mannan groups in *P. boydii* conidia. Conidia recovered from 5-day-old cultures were incubated with concanavalin A conjugated to fluorescein isothiocyanate (Con A-FITC), and examined under fluorescence microscopy. Whereas a few conidia were highly fluorescent (asterisks) and some others were faintly labeled (arrows), the majority exhibited labeling exclusively on their release scar (**A**) as demonstrated by examination of the same field by phase-contrast microscopy (**B**). In (**C**) and (**D**), conidia incubated with a large excess of the inhibitor methyl α-D-mannopyranoside prior labeling with FITC-Con A showed no fluorescence, thus attesting the specificity of the labeling.

Spores from 5-day-old cultures were incubated with fluorescent lectins (Con A, WGA and PNA) in order to detect mannose/glucose, GlcNAc and galactose residues, respectively. No fluorescence was observed at the cell wall surface after incubation with FITC-WGA, which only bound to the scar region that appears at the basis of the conidia after their release (data not shown). In contrast, fluorescence of conidia labeled with Con A-FITC was highly heterogeneous. While a few conidia were intensely labeled (asterisks) and some others faintly marked (arrows), the majority exhibited labeling exclusively on their release scar (Figure 1A and B). Inhibition assays with methyl α-D-mannopyranoside confirmed the specificity of labeling (Figure 1C). No fluorescence was observed after incubation with FITC-PNA (results not shown).

### Effect of the Age of Culture on Cell Surface Properties of Conidia

**Figure 2:**
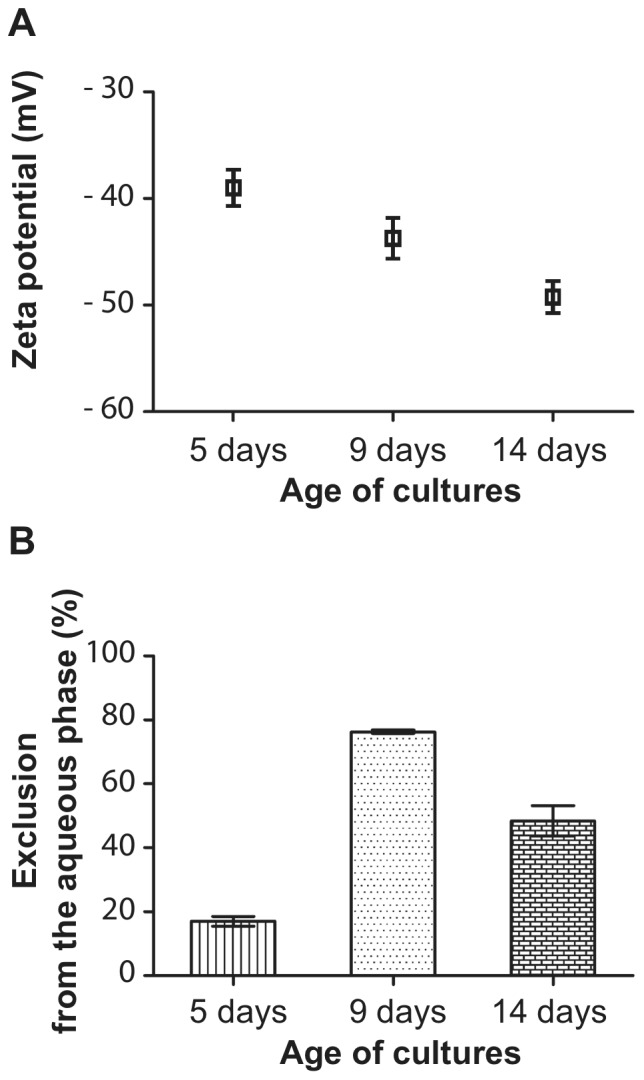
Influence of the age of the cultures on the physical surface properties of conidia. Surface electrostatic charge (**A**) and cell surface hydrophobicity (**B**) of conidia recovered from 5-, 9-, and 14-day-old cultures were significantly different among the three groups with a *p*<0.01 for (A) and *p*<0.001 for (B).

Heterogeneity of the labeling with Con A-FITC suggested maturation of the conidia with the ageing culture. To confirm this hypothesis, we first studied the impact of age of culture on the cell surface properties which reflects the entire composition of the cell wall. Conidia were thus recovered from 5-, 9- and 14-day-old cultures and their surface hydrophobicity and electrostatic charge were measured (Figure 2). Conidia exhibited a high electronegative charge that increased overtime, from −39.0 mV on day 5 to −49.3 mV on day 14 (*p*<0.05). Similarly, the surface hydrophobicity of spores markedly increased after 5 days of culture as shown in Figure 2B (*p*<0.001). The difference in CSH between conidia from 9- and 14-day-old cultures was not significant according to Dunn’s post-hoc test.

### Melanin Detection and Changes in the Melanin Content of the Conidial Wall with the Age of Cultures

#### DHN-melanin in *P. boydii*

**Figure 3:**
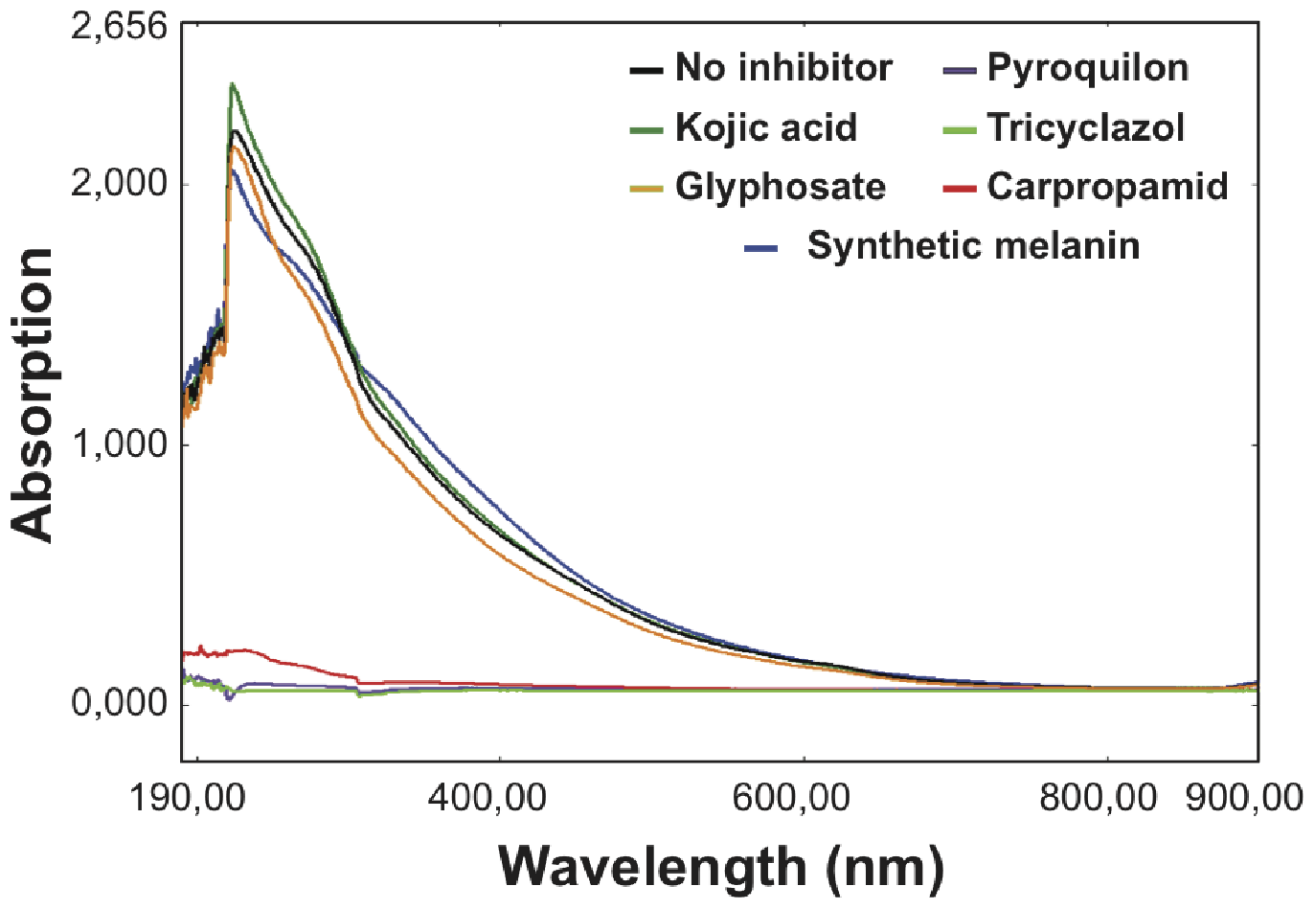
UV-visible spectrophotometry of melanin extracts. Melanin extracts from control conidia or conidia recovered from cultures grown in the presence of DOPA-melanin (kojic acid or glyphosate) or DHN-melanin (pyroquilon, tricyclazol or carpropamid) inhibitors were examined under UV-visible spectrophotometry. A spectrum similar to synthetic melanin (0.05 mg/ml) was obtained only for extracts from control conidia or conidia produced with DOPA-melanin inhibitors.

In order to identify the type of melanin synthesized in *P. boydii,* inhibitors of DOPA- (tropolone, kojic acid and glyphosate) or DHN- (tricyclazol, pyroquilon and carpropamid) melanin synthesis pathways were added to the culture medium and melanin was extracted from conidia after 9 days of incubation of the cultures. Melanin content in these extracts was investigated by UV-visible spectrophotometry over a wide range of wavelengths from 190 nm to 900 nm (Figure 3). Strong absorption was detected in the UV region for conidia taken from cultures grown with or without DOPA-melanin inhibitors, with an absorption maximum at about 226 nm, the same wavelength at which maximum absorption of synthetic melanin occurs. Conversely, extracts of conidia taken from cultures grown in the presence of DHN-melanin inhibitors had no detectable absorption in the UV region, which indicates that only DHN-inhibitors were able to suppress the synthesis of melanin. These results were in accordance with the color of conidia that turned from dark brown to cream color upon incubation with DHN-melanin inhibitors.

**Figure 4:**
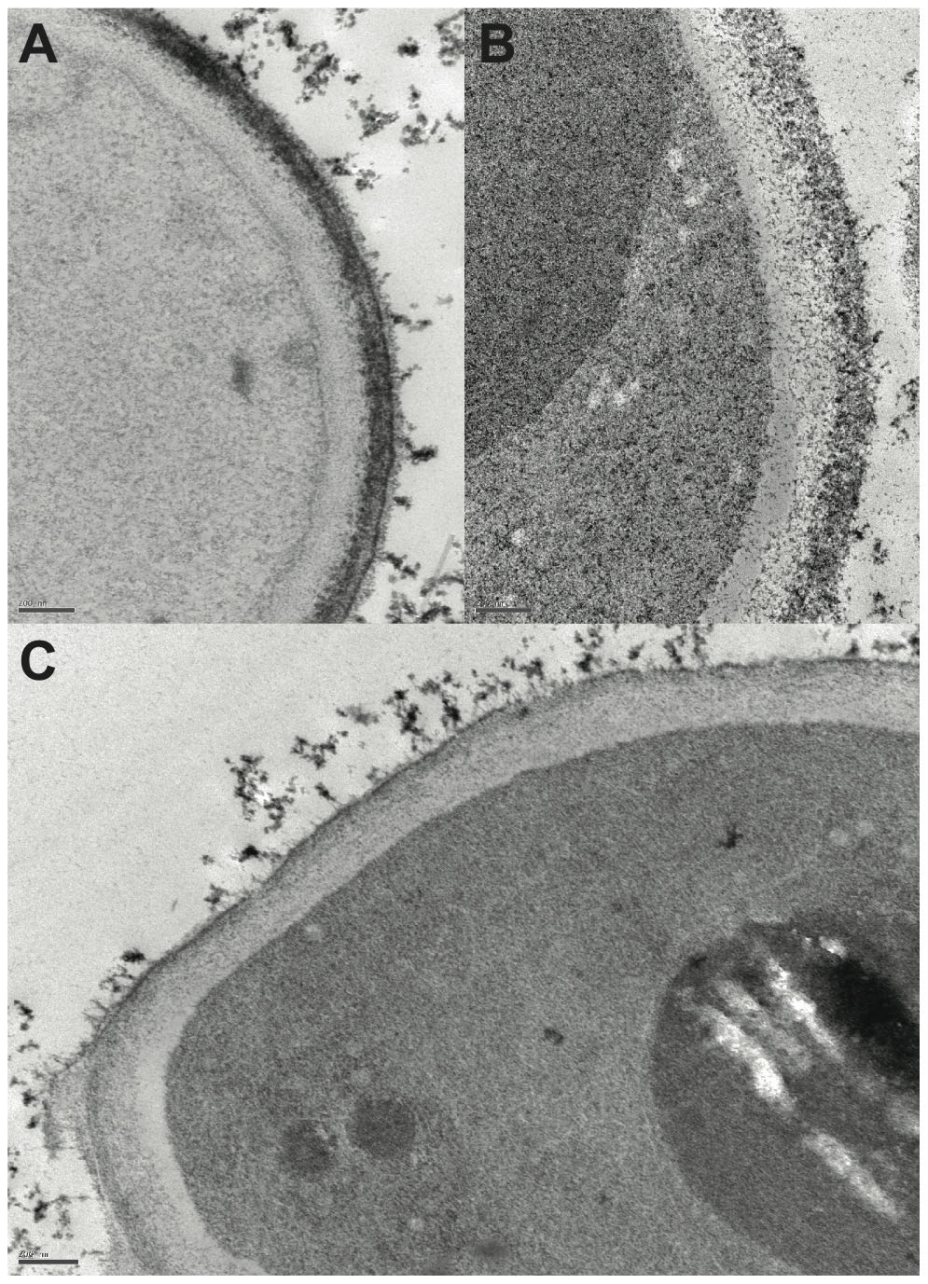
Transmission electron microscopy examination of conidia from cultures grown with or without melanin inhibitors. Control conidia (**A**) or conidia recovered from cultures grown with the melanin inhibitors glyphosate (**B**) or pyroquilon (C) examined by transmission electron microscopy (scale bar = 200 nm).

The effect of melanin inhibitors on the structure of the cell wall in conidia was also examined in TEM (Figure 4). The outer electron-dense cell wall layer became markedly thinner, fragmented and sometimes detached for conidia recovered from cultures grown in the presence of pyroquilon in comparison to the thick uniform outer cell wall layer of control spores or spores recovered from cultures with glyphosate.

#### Sequencing of genes involved in the DHN-melanin biosynthesis pathway

**Figure 5:**
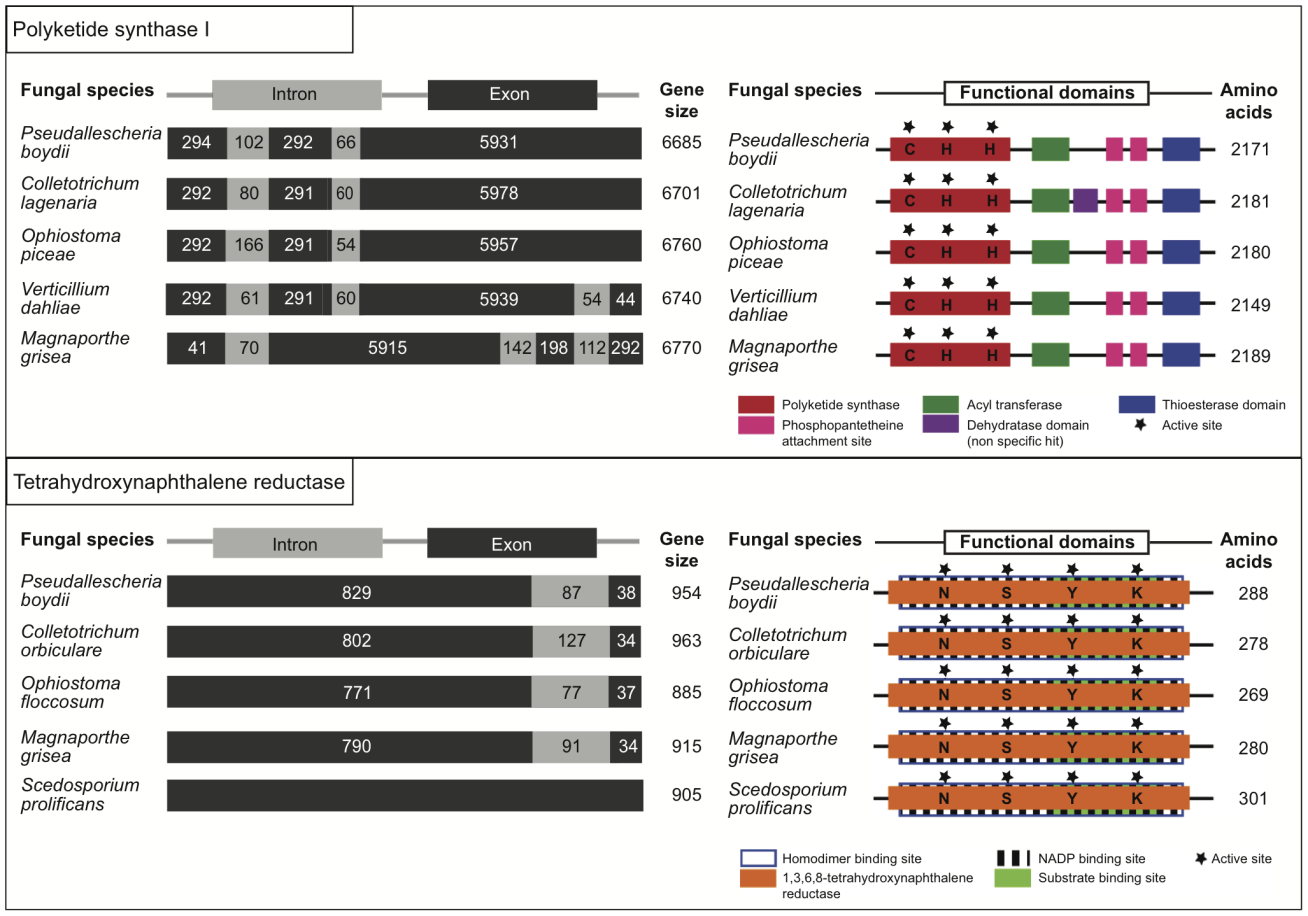
Analysis of *PKSI* and *4HNR* gene sequences and predicted protein sequences. Alignment of protein and nucleotide sequences for polyketide synthase type I of the fungi (Accession number): *Pseudallescheria boydii* (KC440182, this work), *Colletotrichum lagenaria* (BAA18956.1), *Verticillium dahliae* (EGY13508.1), *Magnaporthe oryzae* (ELQ39536.1), *Ophiostoma piceae* (ABD47522.2). Alignment of protein and nucleotide sequences for tetrahydroxynaphtalene reductase of the fungi (Accession number): *Pseudallescheria boydii* (KJ549637, this work), *Colletotrichum orbiculare* (AB661336), *Ophiostoma floccosum* (AF285781), *Magnaporthe grisea* (AY846877), and *Scedosporium prolificans* strain 3.1 (JX861395). Protein functional domains were predicted by conserved domain search in NCBI.

The search for enzymes in the synthesis pathway of DHN-melanin lead to the identification of genes encoding polyketide synthase type I (*PKSI*) and tetrahydroxynaphtalene reductase (*4HNR*) (Figure 5). Genes were sequenced after multiple sequence alignment of homologous genes in other taxonomically close fungi. Function of the corresponding proteins was confirmed by identification of predicted functional domains after translation. Similarly to *Colletotrichum lagenaria* and *Ophiostoma piceae*, *PKSI* gene in *P. boydii* had 2 introns, while homologous genes in *Verticillium dahliae* and *Magnaporthe oryzae* had 3 introns. As for the *4HNR* gene, *P. boydii, Colletotrichum orbiculare, Ophiostoma floccosum and Magnaporthe grisea* all shared one short intron unlike *Scedosporium prolificans* that lacked any. Expression of these genes has also been confirmed by analysis of mRNA extracts from conidia (results not shown), however attempts to perform quantitative real time-PCR failed due to the incapacity to normalize the expression of *PKSI* and *4HNR* genes in our experimental conditions. Four reference genes were chosen based on the work of Fang and Bidochka [32] and Raggam *et al* [33], *i.e.* beta-tubulin, tryptophan biosynthesis enzyme, actin and glyceraldehyde 3-phosphate dehydrogenase, but all showed high delta Ct values when conidia from 5-day-old cultures were taken as reference.

#### Comparison of melanin extracts with UV-visible spectrophotometry

**Figure 6:**
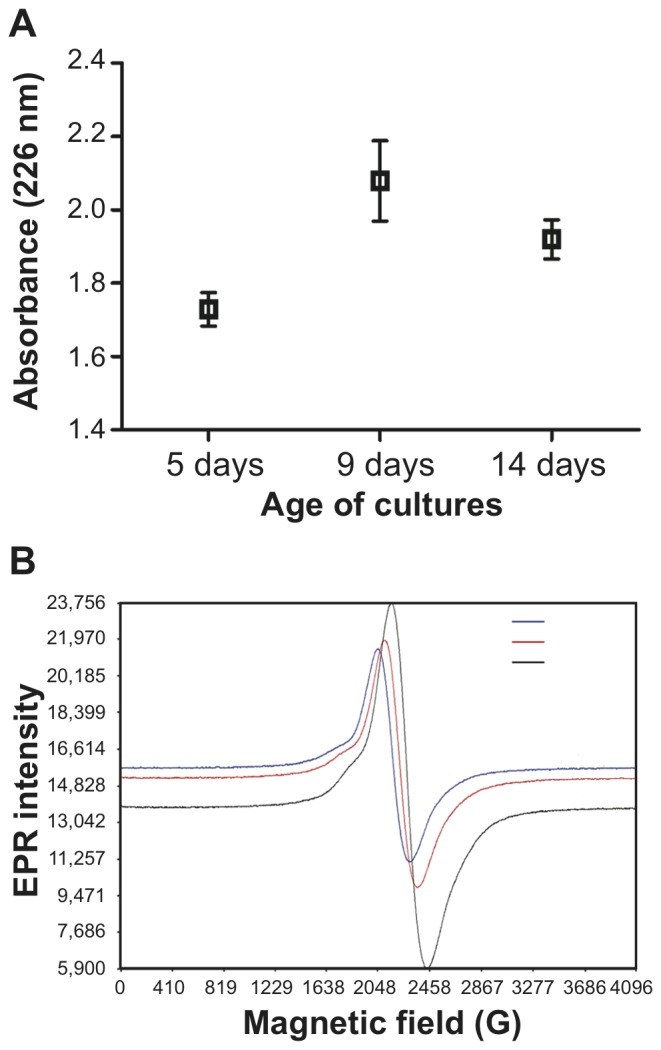
Changes in melanin amount in conidia as the age of culture progresses. Melanin amount in the conidial wall changed with the age of culture as revealed by UV-visible spectrophotometry (**A**) or electron paramagnetic resonance (**B**). (**A**) The mean rank of the quantity of melanin extracted from conidia was significantly different among cultures of different age (*p*<0.05). (**B**) EPR spectra of *P. boydii* conidia taken from cultures of different age (5, 9 and 14 days). The spectra were recorded at a microwave power of 30 db. Spectra are representative of three independent experiments.

Melanin was extracted from conidia recovered from 5-, 9- and 14-day-old cultures, and the maximum absorbance at 226 nm was recorded for all samples analyzed in triplicate (Figure 6A). The mean ranks of absorption were significantly different among the three groups of conidia: melanin content in extracts from conidia increased after 5 days of culture (*p*<0.05). The mean absorbance values obtained for melanin extracts of conidia taken from 9- and 14-day-old cultures were not significantly different according to Dunn’s comparison test (post-hoc analysis).

#### EPR spectroscopy comparison of *P. boydii* conidia taken from cultures of different age

Conidia taken from cultures of different age showed a diminishing EPR signal intensity as the age of culture increased (Figure 6B). Experiments were repeated three times and similar regressions were obtained each time. A decrease in the EPR signal intensity was also found with a wild-type strain of *Aspergillus fumigatus* (CBS 113.26) when conidia isolated from 3- or 8-day-old cultures were compared (data not shown). No EPR signal was observed for PBS buffer alone, or with conidia from a mutant strain of *A. fumigatus* (strain IHEM 9860) blocked in the early steps of melanin synthesis [27].

#### Melanin masks mannose-containing glycoconjugates and affects the electrostatic charge of the conidial surface

**Figure 7:**
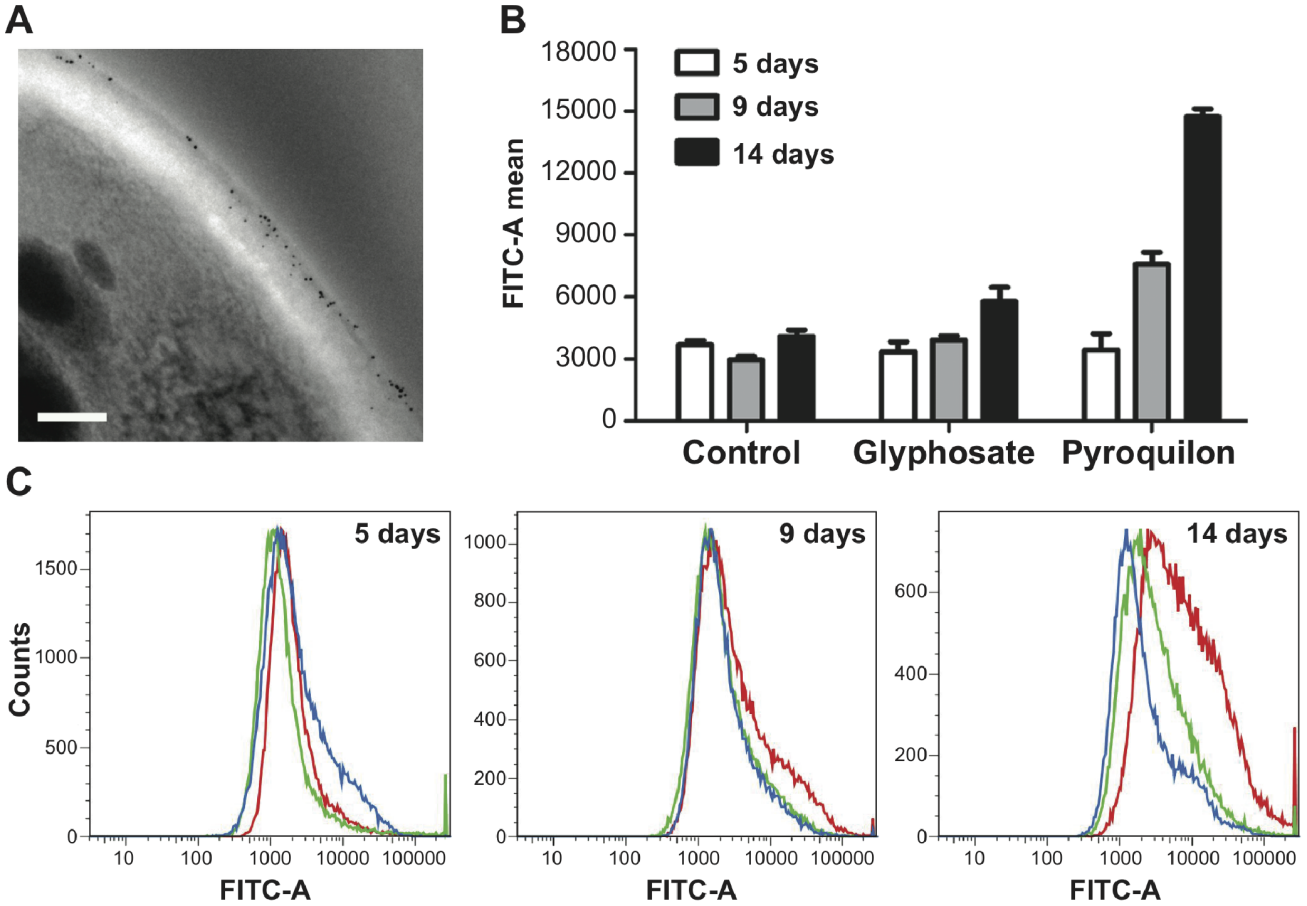
Repercussion of increased melanin synthesis on the surface labelling of conidia with concanavalin A. (**A**) Trasmission electron microscopy showing conidia labeled with gold-conjugated concanavalin A (Con A; 5-nm gold particle). Scale bar = 200 nm. (**B,** **C**) Quantitative analysis by flow cytometry of fluorescence at the surface of conidia treated with fluorescein isothiocyanate (FITC)-ConA. (B) Comparison of mean specific fluorescence intensity of conidia recovered from 5-, 9-, or 14-day- old cultures grown with or without glyphosate or pyroquilon. Contrary to conidia with glyphosate treatment, conidia with pyroquilon treatment showed a significant increase in labelling with respect to their counterparts from 9- or 14-day-old control cultures grown without any melanin inhibitor (*p*<0.001). Specific fluorescence was obtained after normalizing against the fluorescence in conidia untreated with FITC-ConA or conidia treated with methyl α-D-mannopyranoside prior to the addition of FITC-Con A. (**C**) Fluorescence frequency distribution histograms: the blue-lined histogram represents the relative signal of conidia from cultures grown without any melanin inhibitor, whereas the red- or green-lined histograms represent the relative signal of conidia treated with pyroquilon or glyphosate, respectively (number of fungal cells in the y-axis versus relative fluorescence intensity in the x-axis expressed as arbitrary units on a logarithmic scale).

Conidia recovered from cultures containing the DOPA-melanin inhibitor glyphosate were incubated with FITC-conjugated Con A. No significant changes were observed by flow cytometry in the mean fluorescence intensity of these conidia compared to cells from control cultures of the same age (*p*>0.05). In contrast, conidia recovered from cultures grown in the presence of pyroquilon showed a significant increase (*p*<0.001) in fluorescence intensity as the age of culture progressed suggesting an increase in the amount of mannose-containing glycoconjugates in the conidial wall (Figure 7).

**Figure 8:**
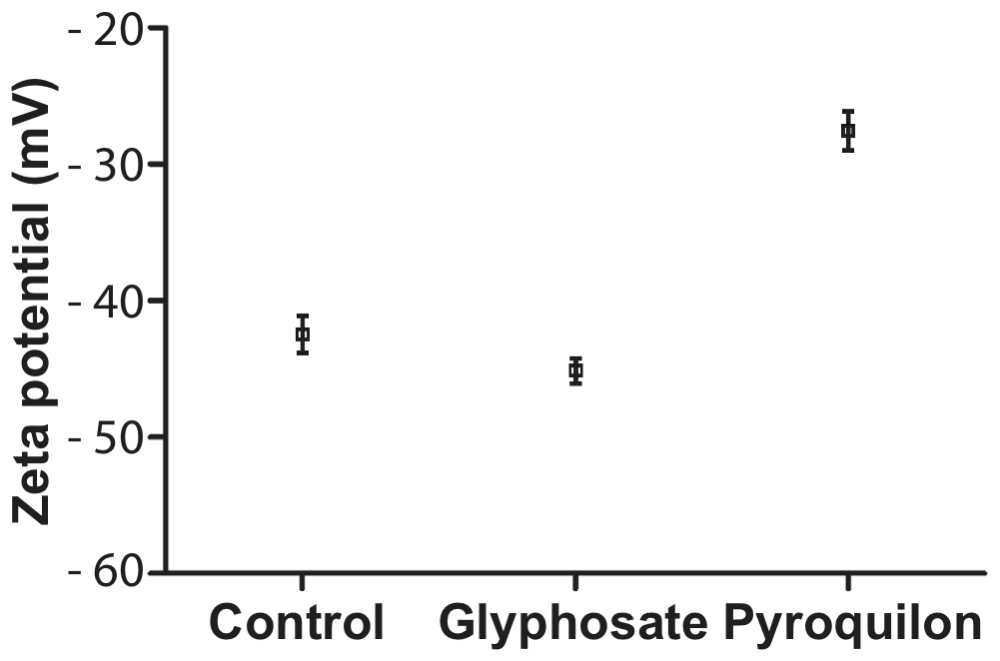
Changes in the physical properties of the conidial surface induced by melanin inhibition. The surface electronegativity of conidia recovered from cultures grown with pyroquilon was markedly reduced with respect to conidia recovered from control cultures or cultures grown with glyphosate (*p = *0.0010).

The presence of melanin is commonly known to affect the surface electrostatic charge in fungi [34]. In order to test this, conidia were recovered from 9-day-old cultures with or without melanin inhibitors. Conidia taken from cultures with pyroquilon showed a marked decrease (*p* = 0.001) in the surface electronegative charge with respect to conidia taken from cultures with glyphosate or control cultures without any inhibitor (Figure 8).

### Absence of Rodlets on Conidia of *P. boydii*

**Figure 9:**
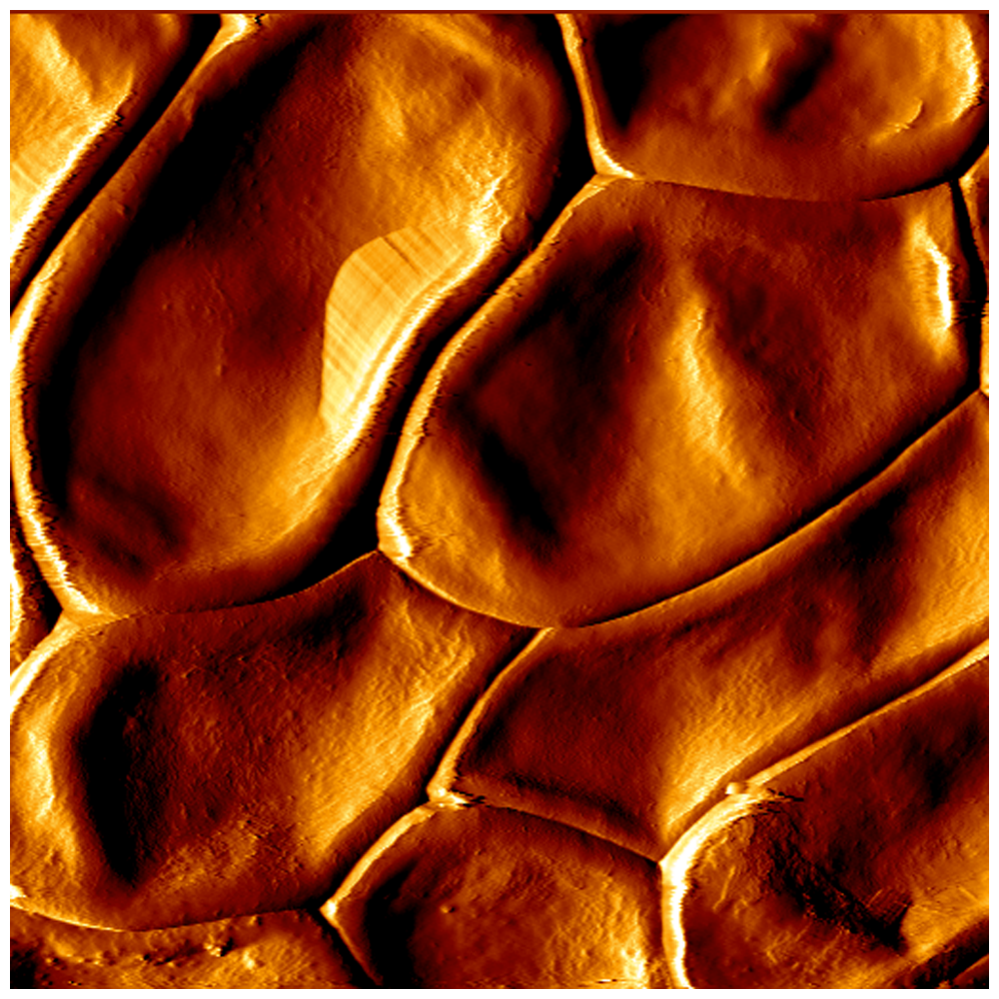
Atomic force microscopy image of the surface of *P. boydii* conidia. Conidia recovered from 9-day-old cultures examined by AFM in the tapping mode showed a smooth surface without any rodlet layer of hydrophobins (10 µm×10 µm image).

As illustrated in Figure 9 for conidia taken from 9-day-old cultures, AFM analysis of conidia from 5-, 9- or 14-day-old cultures showed the absence of rodlet layer at the cell surface whatever the age of culture was (data not shown).

## Discussion

Knowledge of the chemical composition and structural modifications of the fungal cell wall is crucial to understanding how infectious propagules interface with host tissues during early the stages of morphogenesis and pathogen establishment. To date, our knowledge of *P. boydii* is largely restricted to taxonomical studies and medical case reports. Interest in the molecular genetics, biochemistry and cellular physiology of this fungus is still in its infancy and so the present study set out to investigate cell wall modifications during maturation of the *P. boydii* conidium, the infectious propagule of this emerging human pathogen. Our interest in studying conidial maturation came from the observation that conidia from the same culture labeled with mannose/glucose-specific lectin Con A showed an unexpected heterogeneity in the surface fluorescence intensity from one spore to another. It is well known that the cell wall in *P. boydii* comprises peptidorhamnomannan as the main structural component,and this molecule was shown to activate TLR4 and to mediate adherence to HEp2 epithelial cells [21], [22]. The observed variability of fluorescence between cells could not be explained by swelling, as is the case with *Aspergillus fumigatus*, since conidia that were intensely labeled with FITC-conjugated Con A were of a similar size to unstained conidia. Likewise, thickness of the outer cell wall layer was unaltered and no cytoplasm vacuolization was observed. Therefore, to explain the variability among conidia taken from the same culture, we hypothesised that they were undergoing maturation. To test this, we investigated variations in cell surface physical properties in conidia taken from 5-, 9- and 14-day-old cultures. Examining surface physical properties provides a general overview of the cell surface reflecting the properties of its components and highlights the changes in the biochemical composition of the cell wall. Comparison of conidia showed a marked increase after day 5 in both CSH and the surface electronegative charge. Although the ensemble of cell wall components are implicated in giving conidia their surface physical property signature, a prominent role of certain components, such as melanin on the surface electrostatic charge for example, has previously been demonstrated in other fungal species like *A. fumigatus* [27] and *C. neoformans* [35], [36]. Wang *et al.* [37] showed that melanization of *C. neoformans* increases in an approximately linear rate over the course of a 14-day growth period. Nosanchuk and Casadevall [35] also performed a time course analysis of melanization and modification in the surface charge of conidia. They found that an increase in the electronegative charge in late stationary phase paralleled a progressive melanization in *C. neoformans* cells. Similarly, in *Trichoderma* species, Pokorny *et al.* [38] found that the activity of laccase, an enzyme responsible for the final step of DOPA-melanin synthesis, varied in conidia according to the age of cultures. Indeed, this activity reached a maximum in conidia taken from ≈14-day-old cultures. In *P. boydii*, no previous studies were available about melanin synthesis. Therefore we investigated the type of melanin produced by conidia by incorporating DHN- or DOPA-melanin inhibitors in the culture medium. A clear difference could be seen in the color of conidia isolated from cultures grown in the presence of DHN-melanin inhibitors after 9 days of incubation. UV-visible spectrophotometry analysis of melanin extracts confirmed that DHN-melanin is a major pathway for the production of melanin in *P. boydii*. Enzymes involved in the DHN-melanin synthesis pathway were also found in mRNA extracts. Melanin amount in extracts from conidia progressively increased after day 5. Our experiments demonstrated that melanin synthesis influences the ultrastructure of the condial wall and that its presence affects the zeta potential of cells. Inhibition of melanin synthesis by the addition of pyroquilon led to a marked decrease in the electronegative charge of the conidial surface.

Melanins naturally contain unpaired electrons that can be detected by EPR as a free radical signal [39]. Synthesis intermediates, capable of generating free radicals through a reversible reaction, polymerize to form melanin polymers. Although phenolic subunits have in some instances been discovered in melanin polymers, the exact arrangement of these subunits in the polymer remains unknown [40]. Numerous studies have previously associated damage to melanin, by ionizing radiation for example, to the production/liberation of free radicals and an increase in the EPR signal intensity suggesting a depolymerization event. On the other hand, melanin is also known for its antioxidant properties. Melanin scavenges reactive oxygen species (ROS), such as singlet oxygen hydroxyl radicals and superoxide anions, thus conferring protection against oxidative stress inside human host cells. The balance between the intrinsic anti-oxidant and pro-oxidant properties of melanin determines it redox status and its degree of polymerization or the molecular weight of the melanin polymer [41], [30]. In this study we demonstrated that the increasing quantity of melanin in *P. boydii* is accompanied by a decrease in EPR signal intensity in conidia, which implies that melanin tends to polymerize with time and potentially gains a higher antioxidant activity.

Finally, to conclude on our primary observation that conidia did not show a homogeneous labeling with fluorescent Con-A lectin, we demonstrated in this study that the time-dependent increase in melanin amount at the conidial surface during maturation of the spores progressively masks the cell wall mannose-containing glycoconjugates, thus diminishing the accessibility of the conidial surface components to the lectin.

Melanin and hydrophobins provide efficient systems to evade recognition by the immune system and counteract the deleterious effect of reactive oxygen species. In *P. boydii*, we didn’t find any rodlet layer on the surface of conidia with AFM analysis irrespective of culture age. However, the absence of such structures does not mean that hydrophobins are absent at the cell surface. In *Aspergillus fumigatus* there are different class I hydrophobins but unlike rodAp, rodBp does not form rodlets. Furthermore class II hydrophobins, which are frequent in the order *Sordariomycetes* (to which *P. boydii* belongs), were never seen to form rodlets at the surface of fungal entities [42]. Identifying and sequencing hydrophobins by nucleotide homology was not possible since protein sequences in fungi have very poor homology except for eight cystein residues and few other amino acid residues characteristic of class I or class II hydrophobins.

## Conclusion

The conidial wall is a highly dynamic structure and is subject to changes and modifications with culture age. These findings have important biological consequences since the fine balance between immunogenic molecules at the cell surface, such as cell wall carbohydrates, versus molecules that mask these such as DHN-melanin, needs to be taken into consideration for future studies aimed to understand fungal pathogenicity. All these components conspire to influence the adherence process and the evasion from host immune defenses. Culture conditions should be strictly standardized for all studies concerned with the adherence mechanisms, evasion from host immune defenses or virulence in animal models. Moreover, elucidation of the melanin biosynthesis pathway in *P. boydii* and identification of two of the genes involved offer new opportunities to establish the role of melanin in protecting the fungus against stresses it may be exposed to in the host or in the natural environment. For instance,species of the *P. boydii/S. apiospermum* complex are ubiquitous fungi that are particularly common in highly polluted soils and waters, and melanin at the conidial surface would likely be required to protect spores against toxic agents such as aromatic compounds or heavy metals. Fungicides targeting the synthesis of DHN-melanin may be useful to reduce the dispersal of these pathogens.
